# A Treatise on Foreign Bodies in Surgical Practice

**Published:** 1880-05

**Authors:** 


III.—A Treatise on Foreign Bodies in Surgical Practice. By Alfred Poulet, M.D., Adju. Surgeon Major, Inspector of the School for Military Medicine at Val-de-grace. In two Volumes, constituting part 3d and 4th.

